# Costs of syringe vending machines in Tbilisi, Georgia

**DOI:** 10.1186/s12954-023-00829-3

**Published:** 2023-08-02

**Authors:** Josephine G. Walker, Irma Kirtadze, Mzia Tabatadze, Peter Vickerman, David Otiashvili

**Affiliations:** 1 Population Health Sciences, University of Bristol, Bristol, UK; 2 Alternative Georgia, Tbilisi, Georgia; 3 Ilia State University, Tbilisi, Georgia

**Keywords:** Harm reduction, Needle and syringe provision, People who inject drugs, Caucacus

## Abstract

**Background:**

Syringe vending machines (SVM) can improve access to sterile injecting equipment, but they have not been widely implemented or evaluated. We evaluate the cost of SVM installed between July 2019–December 2020 in Tbilisi, Georgia.

**Methods:**

The SVM were stocked with several kit types, including injecting equipment for opioid or stimulant users, naloxone, male and female condoms, and pregnancy tests. We gathered financial data from the project to estimate fixed (staff time, start-up costs, equipment, running costs, and consumables) and variable (harm reduction kits) costs. We calculated the full cost of the SVM intervention, cost per user, cost per additional syringe accessed by SVM users, and cost per kit distributed (2020 Euros).

**Results:**

SVM access cards were issued to 1132 users, and 29,238 kits were distributed through SVM, total cost €204,358. Staff costs were 51% of total, consumable costs 28%, equipment 10%, and start up, recurrent costs, and overheads 5% or less each. Opioid and stimulant kits were most accessed (35% and 32% of total). Cost per user was €66/year, and cost per transaction €7, of which €5 fixed costs and €2 variable. If monthly transactions increased from the average of 1622/month to highest monthly usage (4714), fixed costs per transaction would decrease to < €1. It cost €0.55 per additional syringe accessed/user/month.

**Conclusions:**

This study provides evidence for governments about the cost of SVM, a novel harm reduction intervention. This is particularly relevant where Global Fund is withdrawing and harm reduction services need to be incorporated into national budgets.

**Supplementary Information:**

The online version contains supplementary material available at 10.1186/s12954-023-00829-3.

## Background

Georgia has been identified as a country with one of the highest prevalences of injecting drug use in the world [1, 2]. Estimates suggest there are approximately 52,500 people who inject drugs (PWID) in Georgia, making up approximately 2.2% of 18–64 year olds, compared to a global average of 0.33% (0.21–0.49%) [1, 3].

Harm reduction programs, including needle and syringe programs (NSP) are an effective way to reduce injecting risk behaviours such as syringe re-use and sharing, and the risk of HIV and Hepatitis C virus (HCV) transmission among PWID [4]. The greatest prevention benefits are found when NSP and opioid substitution therapy (OST) are available in combination and at high coverage [5]. A Cochrane review found that high NSP coverage in Europe was associated with a 76% (95% confidence interval (CI) 38–91%) reduction in HCV acquisition risk [6]. Data from the Georgia Harm Reduction Network (GHRN) estimate that 3.9 million syringe kits were distributed in 2019, reaching more than 35,000 PWID [7]. Injection frequency among PWID has been found to be lower in Georgia than other countries, with only 2% of PWID injecting daily or more [8], a mean of 14.2 (95% CI 12.3–16.1) injections in the last month among PWID not in contact with harm reduction services [9], with an average number of days injecting per month between 13.8–17.7 [10]. A systematic review in 2017 categorized Georgia as having low OST and NSP coverage [5], however access to OST and NSP have increased since then, to approximately 60% coverage of OST and 67% coverage of NSP (defined as any contact with NSP programs within a year) [11]. A recent bio-behavioural survey found 78.7% of PWID reported always using new sterile needles in the last month, with the main sources of sterile needles from drugstores (77.3%) and NSP (53.0%) [12]. In addition to NSP and OST, in Georgia, harm reduction programs are allowed to distribute naloxone which can prevent overdose-related deaths [9].

As with many other countries in Eastern Europe and Central Asia (EECA), Georgia is in the process of transitioning from full funding of NSP and other harm reduction programs by The Global Fund to Fight AIDS, Tuberculosis, and Malaria (GF) to national funding [13]. As GF support is withdrawn, there is a critical need to adopt innovative approaches to HIV/HCV prevention to optimize resource allocation and sustain harm reduction programs going forward. In 2021, when still fully funded by GF, the harm reduction program supported 14 fixed sites in 13 cities and 9 mobile units providing NSP, infectious disease testing, naloxone distribution, information and education, and referral to health services [14].

The addition of syringe vending machines (SVM) to the harm reduction provision could supplement standard NSP, including providing access to sterile injecting equipment outside of harm reduction site opening hours, reaching groups who are harder to reach, such as younger PWID, women, and those in geographical areas without fixed or mobile sites. Previous studies in Western Europe, Australia, and New Zealand have demonstrated the effectiveness of SVM in supplementing existing NSP services [15–21]. A respondent-driven sampling study of PWID not currently using harm reduction services conducted in Tbilisi in 2018 found that 97% of respondents said they would personally use SVM [9]. In addition to sterile injecting equipment, the SVM machines can supply other injecting paraphernalia, condoms, naloxone, HIV self-tests, and other health supplies useful to the target groups [22].

There is very little previous published data on the cost or cost-effectiveness of syringe vending machines. One study reports that vending machines will be more cost-effective than fixed or mobile harm reduction sites due to the high cost of personnel associated with standard harm reduction services, and the relatively low proportion of total costs for syringes [17]. In particular, costs could be significantly reduced compared to providing a staffed harm reduction service 24 h per day [15].

We aim to estimate the costs of a real-life implementation of SVM to provide crucial evidence for informing national and sub-national governments about the potential cost of this harm reduction intervention. This is particularly relevant for those countries where governments will need to take on funding harm reduction programs as aid funding, such as from the Global Fund, is withdrawn.

## Methods

Addiction Research Center Alternative Georgia (ALTGEO) in partnership with the National Center for Disease Control and Public Health (NCDC) and GHRN conducted an implementation trial of syringe vending machines in Georgia from 2018 to 2021 [9, 22, 23]. In this study, we calculate the full economic cost of the SVM intervention overall, and per machine, based on financial data provided by the project, and calculate the cost per user, cost per transaction, and cost per kit distributed.

### Overview of intervention

The SVM project began in April 2018. Workshops were conducted with local harm reduction providers to discuss the project and establish a community advisory board for the project. Mixed methods research was conducted to understand acceptability and willingness to use SVM, through focus group discussions with existing NSP clients and service providers, respondent-driven sampling surveys among PWID who do not currently use NSP [9], and individual interviews with health authorities. Prior to SVM installation, harm reduction providers and SVM installation/maintenance staff received training.

A total of 10 SVM were installed between July 2019–June 2020 in Tbilisi, Georgia, with each pair of machines affiliated to five different harm reduction providers. The trial was designed with a stepped-wedge format, such that machines were installed at regular intervals throughout the study period [23]. The first eight machines are associated with four organizations that focus on harm reduction for PWID (Hepa+, installed July 2019; New Vektor, installed October 2019; New Way, installed January 2020; and Akeso, installed June 2020), with the final pair of machines associated with an organization that works with men who have sex with men (Equality Movement, installed October 2020). Harm reduction providers distributed access cards to their users, including secondary cards to distribute to peers not in contact with harm reduction services. Users were able to look up the locations of all SVMs through an online portal [22].

Each machine was also associated with a local pharmacy, with a subset of distribution slots available for harm reduction kits that are visible and accessible only to those with cards distributed by harm reduction providers, and remaining slots for materials of interest to the general population provided by the pharmacy. A variety of harm reduction kits were available through the machines, including separate packs for opioid or stimulant users, overdose prevention, female and male condoms, pregnancy tests, and informational brochures. Kits with injecting equipment for opioid or stimulant users were designed to provide syringes and paraphernalia for a group of up to four people injecting together. This was based on focus group discussions and injecting group sizes reported by NSP users (mean group size of 3.85 with 62% reporting injecting in a group of 3–5 people) [24] and through a peer-driven intervention study (mean group size 3.31 with 68.8% reporting a group size between 3–5) [25]. Following establishment of the machines associated with Equality Movement, HIV self-test kits and lubricant were also added to the machines.

### Costing methods

Full expenditure records from the project between April 2018 and December 2020 were provided and categorized in order to estimate fixed costs (those that remain the same regardless of number individuals reached by the program) and variable costs (linked to machine usage) for project implementation. Fixed cost categories included staff time (project director, data manager, stock manager, accountant, technical staff), start-up costs (such as staff training and machine installation costs), equipment (computers and the vending machines), recurrent costs (utilities and maintenance costs for the machines), and overheads (office running costs). Access cards and harm reduction kits distributed from the machines were calculated as variable costs.

Research-related costs that would not be included in future implementations were identified and excluded. Research costs that were excluded entirely included the community advisory board, focus group and survey costs including some data collection equipment, and publication costs. Proportions of the cost of meetings with the harm reduction providers, staff time, and overheads, were excluded based on proportions of time spent on research versus implementation as reported by key project personnel.

Overhead costs were calculated separately from project expenditure, based on average office running costs over 2015–2020, including utilities, stationery and other consumables, and maintenance. We included an estimated value for renting the office space, which was donated. Based on interviews with project staff, 60% of overhead costs were allocated to the SVM project, with 50% of those allocated costs excluded as purely research related.

The annualized value of purchased equipment was calculated using straight-line depreciation over the expected lifespan of each item, assuming 5 years of usage for computers and 10 years for the vending machines.

The cost of each harm reduction kit was calculated based on the ingredients that are included in each kit. Unit costs of individual consumables within the kit (including different syringe and needle sizes, water for injection, alcohol swabs, naloxone, condoms, pregnancy tests, etc.) were summed to calculate the total cost of each kit type. The unit costs of each item were calculated based on project procurement costs for items provided by the project, or from GF procurement records from July 2019 (first SVM installed) to December 2020 (end of costing period) for items provided by GF. Total costs presented include all project and GF costs; in addition, for each kit type we calculated the proportion of the cost that was from GF versus the project.

All costs are presented as 2020 Euro values, with earlier costs inflated to 2020 values using the EuroStat price index “HICP Euro area 19 countries—annual average indices”. The full costing analysis and details of kit contents are available in Additional file 1.

### Outcome methods

Outcomes of interest were extracted from project data. These included the number of machines, users, and number of transactions or packages distributed, and total vending machine-months in place, accounting for the phased installation process. We also calculated a modified estimate of machine-months in operation, which accounted for the proportion of time when some kits were not available due to stock outs or machines being out of service, which occurred between August and December 2020, due to the COVID-19 pandemic affecting stock acquisition. This accounted for the total number of days each of 7 kit types were unavailable as a proportion of the number of days the machines were available. As the reach of the project was less than expected, with variation in month-to-month usage due to changes in COVID-19 restrictions including lockdowns and curfews [23] we also explored the effect on cost of using a target outcome of 10,000 packages distributed per month, and the number of packages distributed in the peak month of operation.

**Fig. 1:**
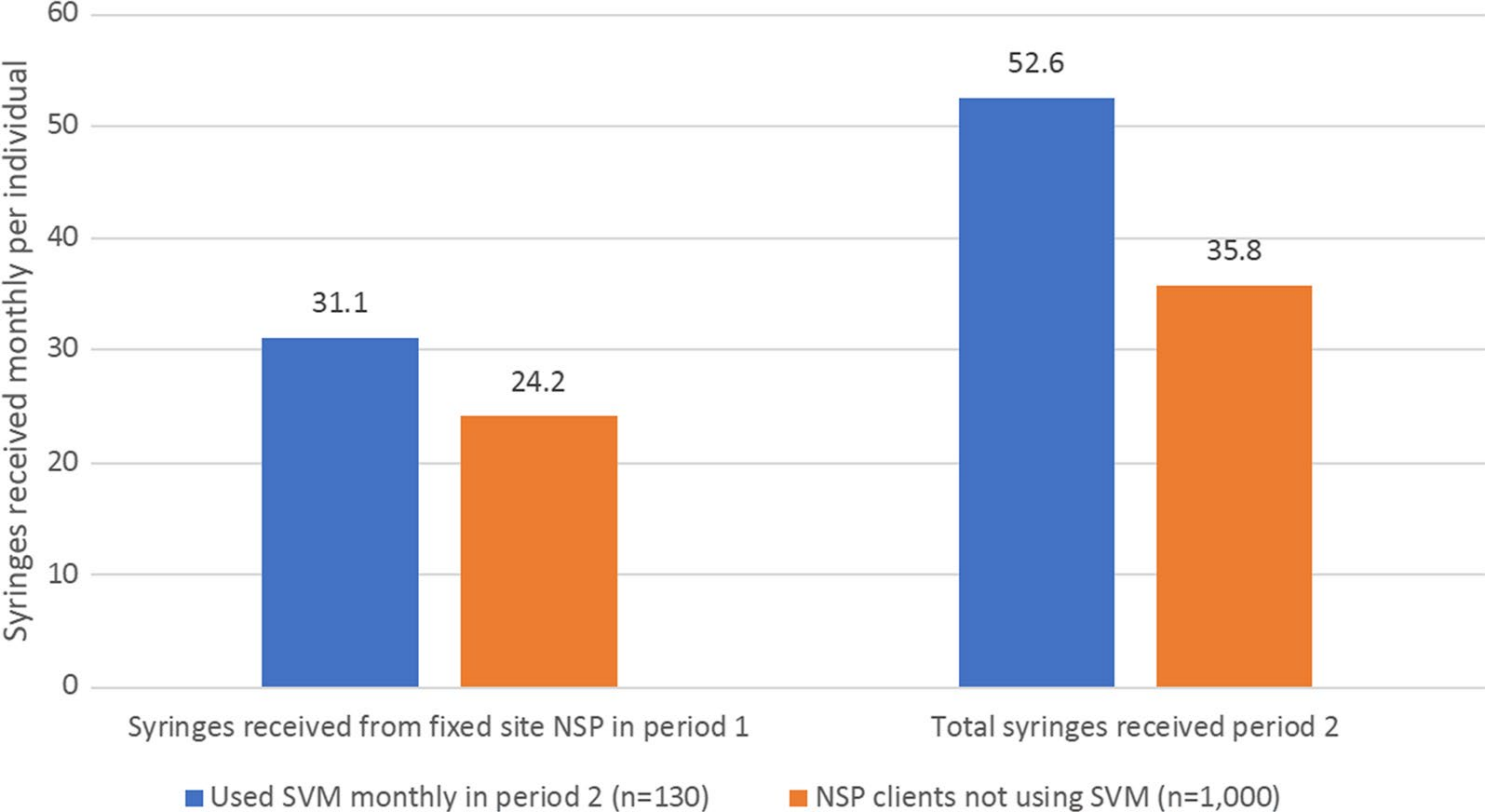
Change in syringes received monthly for NSP users who do or do not access SVM, from data presented in [23]. Period 1 (pre-SVM installation) refers to January–June 2019 and Period 2 (SVM available) is November 2020-April 2021

In addition, we used estimates from the implementation trial of how much the SVM increased syringe access and linked new individuals to harm reduction services [23]. Although all NSP users (both SVM and non SVM users) received more syringes after installation of SVM, SVM users received an additional 9.9 syringes per month compared to non SVM users; this was calculated by comparing the number of syringes received prior to implementation of SVM and after implementation of SVM for clients of NSP who accessed SVM compared to clients of NSP who did not access SVM [23]. Over this period, for NSP clients not using SVM the number of syringes received monthly increased by 11.6 and for SVM users the number of syringes received monthly increased by 21.5, a difference of 9.9 syringes per month (Fig. 1). Only 14 additional people who had not accessed harm reduction prior to the trial went to HIV prevention sites and received harm reduction services after receiving a secondary SVM card [23].

### Cost per user reached and costs per transaction

We calculated the cost per user reached by the SVM, cost per additional syringe accessed, and cost per new person linked to harm reductions services. We also calculated the total cost per machine, transaction, and machine-month, with a breakdown of fixed versus kit consumable (variable) costs. The fixed cost component of the average cost per transaction (excluding the unit cost of the kit itself) was then used to calculate the total distribution cost for each kit type separately, under average, peak, and target transaction levels. Costs and outcomes are not discounted as results are not projected into the future.

## Results

### Outcomes

**Fig. 2:**
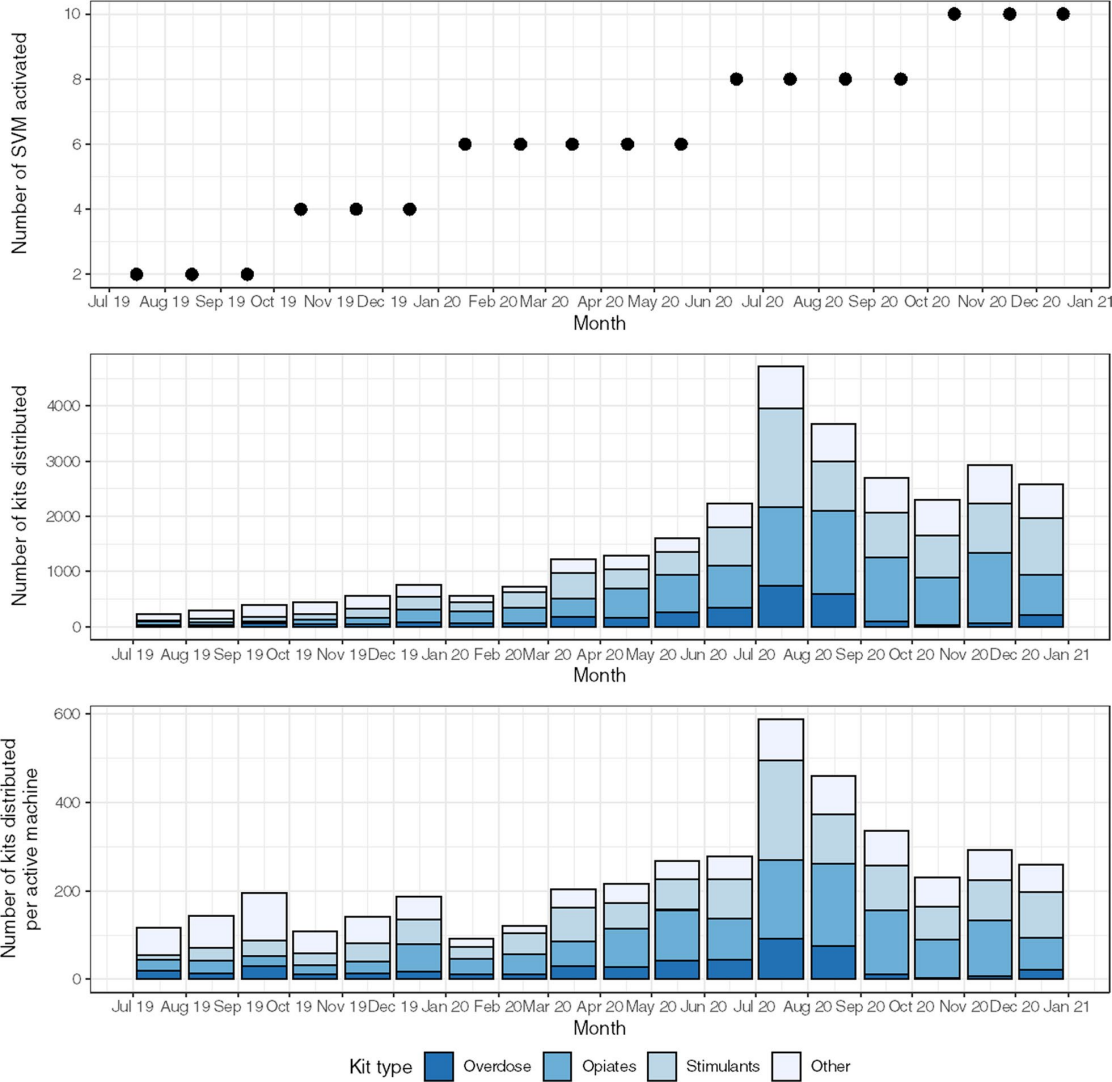
Number of syringe vending machines (SVM) active, total transactions and per SVM by month of study period, for different kit types

A total of 10 SVM were installed, active for a total of 104 machine-months over the study period (July 2019–December 2020). The first machines installed were active for approximately 18 months, with the final two active for only 2 months during the study period. When stock outs are accounted for, the machines were in operation for a total of 96 machine-months. A total of 1132 registered NSP beneficiaries were given SVM access cards, with 226 secondary cards given for peer distribution (total of 1358 access cards distributed). Over the study period, 29,238 kits were distributed through the SVM. In the most active month (July 2020), a total of 4714 packages were distributed in total, as compared to 1622 per study month overall [23] (Fig. 2).

### Total costs

The total cost of implementing the project from April 2018 to December 2020 was €204,358, with an additional €169,135 excluded as research costs. The total cost per month of the project was therefore €6193. The largest component of the cost came from staff costs, at 51%, with consumable costs for the kits making up 28%, equipment 10%, and start up, recurrent costs, and overheads 5% or less each (Table 1). The largest proportion of staff costs was for the data manager (31% of staff costs, see supplementary materials).

**Table 1:** Total cost and proportion attributable to each component

Category	Total project cost	Cost per month	Proportion of total
**Fixed costs (total)**	€147,746.84	€4477.18	72.30%
Staff	€103,435.24	€3134.40	50.61%
Equipment	€19,994.75	€605.90	9.78%
Start-up costs	€4589.73	€139.08	2.25%
Recurrent costs	€8716.82	€264.15	4.27%
Overheads	€11,010.29	€333.65	5.39%
**Variable costs (total)**	€56,611.44	€1715.50	27.70%
All consumables (kits)	€56,611.44	€1715.50	27.70%
**Total fixed and variable**	€204,358.28	€6192.68	100.00%
Excluded costs	€169,135.24	€5125.31	–

The project covered the full cost of the female condoms, pregnancy tests, and information brochure kits, but only 10% of the cost of opioid injecting kits, 11% of male condom kits, and 2% of each of the stimulant injecting kits and overdose prevention packs (naloxone), with the remainder coming from GF funding. The proportion of GF funded kits paid by the project was due to packaging and information brochures included in the packs. The most commonly used kits were the opioid and stimulant kits, making up 35% and 32% of the total kits utilized, respectively, while 14% of kits used were male condoms, and 11% overdose prevention kits. Female condoms made up 4% of distributed kits, pregnancy tests 3%, and information brochures 2%. As a result, items provided by GF contributed 92% of the total distributed kit costs.

The total cost of the project for each of the ten machines was €20,436, with a cost of €1963 for each machine-month, of which €543 went towards harm reduction kits, with the remainder fixed costs, of which 50% were staff costs (Table 2).

**Table 2:** Project cost per machine and per machine-month in place

Category	Average cost per machine installed	Cost per machine-month in place
Staff	€10,343.52	€993.73
Equipment	€1999.47	€192.10
Start-up costs	€458.97	€44.09
Recurrent costs	€871.68	€83.75
Overheads	€1101.03	€105.78
All consumables	€5661.14	€543.88
Total cost	€20,435.83	€1963.33

### Cost per user reached and costs per transaction

The total project cost per user was €180, over a study duration of 33 months, or €5.47 per month. The cost per observed transaction was €7, of which approximately €5 was fixed costs and €2 consumable costs (Table 3). The fixed costs per transaction would be reduced with a higher number of transactions, down to less than €1 if the number of transactions matched those in the peak month of usage, and less than €0.50 if a target of 10,000 transactions per month were reached.

**Table 3:** Project cost per user, and per transaction (observed, peak: assuming 4174 transactions per month as seen in month of highest usage, and target of 10,000 transactions per month)

Category	Cost per user	Cost per transaction (observed)	Cost per transaction (peak)	Cost per transaction (target)
Staff	€91.40	€3.54	€0.66	€0.31
Equipment	€17.67	€0.68	€0.13	€0.06
Start-up costs	€4.06	€0.16	€0.03	€0.01
Recurrent costs	€7.70	€0.30	€0.06	€0.03
Overheads	€9.73	€0.38	€0.07	€0.03
Total fixed costs	€130.56	€5.06	€0.95	€0.45
All consumables	€50.02	€1.94		
Total cost	€180.58	€7.00		

The cost per additional syringe accessed per user per month was €0.55. The cost per additional person (*n* = 14) accessing harm reduction services was €14,597.

The total costs of each harm reduction kit type, including the fixed cost per transaction, the cost of the SVM card per transaction (< €0.01), and the unit costs of each kit are shown in Table 4. The overdose prevention kit is the most expensive, and informational brochure the least expensive kit.

**Table 4:** Cost of distributing each kit type at average, peak, and target transaction numbers

Kit type	With observed cost per transaction	With peak cost per transaction	With target cost per transaction
Pack for opioids	€6.86	€2.74	€2.24
Pack for stimulants	€7.57	€3.45	€2.95
Overdose prevention	€8.95	€4.84	€4.33
Female condom	€6.08	€1.96	€1.46
Male condom	€5.34	€1.22	€0.72
Pregnancy test	€5.32	€1.21	€0.70
Info brochure	€5.10	€0.98	€0.48

## Discussion

This study presents the total costs, and cost per user reached, cost per additional syringe, cost per new access to harm reduction, and cost per transaction of implementing syringe vending machines in Tbilisi, Georgia. Despite challenges of the project running during the COVID-19 pandemic, during which curfews were imposed and equipment shortages experienced, the project was implemented successfully, demonstrating that SVM are acceptable, feasible and effective for improving access to sterile injection equipment for PWID [23]. The total cost to run each SVM was approximately €2000 per month, or €7 per transaction. Increasing the usage of the machines to levels seen in the month of highest usage (July 2020, when there were few COVID-19 related restrictions), would bring transaction costs down to less than €1 plus the cost of each kit.

A previous study of harm reduction costs estimated that the unit cost per client per year to access NSP in Georgia was 396.1 Georgian Lari or $240 (USD) in 2013 [26], which inflates to approximately €191 in 2020. In this study, the cost per user per year was estimated to be €66. Here incremental cost per additional syringe accessed per person was €0.55 per month. This is comparable to the range of 0.18–1.44 2020 USD (approximately 0.16–1.26 in 2020 Euros) estimated as the cost per syringe distributed through NSP in upper-middle-income countries [27]. As injecting kits distributed through SVM included sufficient equipment for four individuals injecting together, the impact of each kit accessed is likely to be much higher compared to standard NSP where syringes are distributed individually, thus improving the cost-effectiveness of the SVM intervention. Furthermore, the machines used in this study were modified to allow for real-time data tracking as required for the evaluation of the intervention [22]. Simpler and cheaper vending machines could be used for future implementations which would reduce the cost of the machines and their maintenance, including by reducing staff costs associated with programming the machines and data management (40% of total staff costs).

The data collection from the machines allowed for a key strength of this study, which is the evaluation of a real-life intervention with thorough record keeping allowing for precise estimation of the costs of the SVM trial. However, this study had several limitations. It was difficult to measure the longer-term impact of SVM leading to linkage of PWID to harm reduction services through distribution of secondary cards. This required additional data reporting from the harm reduction centers which was not done consistently. The estimate that only 14 people accessed services for the first time through SVM results in a high cost per new linkage to services, however we were not able to evaluate the long term impact of this linkage. In addition, the study implemented a limit on the number of 2 injection kits which could be accessed from the vending machine per card within each calendar day, and the number of users who reached this limit was not able to be calculated. This could have led to an underestimate of how much individuals would prefer to access harm reduction materials from SVM. In addition, we have not explored the differences in usage between machines, although machine location might play a role in the effectiveness of an SVM intervention. Machine usage varied over time, and due to the timing of the study including COVID-19 related curfew restrictions in Tbilisi, this is likely to have limited the uptake of SVM usage.

## Conclusions

Despite the challenges of implementation of a new intervention during COVID-19 restrictions, the SVM program was effective at improving access to supplies for those who were already accessing harm reduction services [23].

These costing results will be important for decision makers considering the relative cost of different harm reduction interventions as national programs take on costs previously funded by GF programs. In addition, they can be incorporated into future modelling exercises to evaluate the cost-effectiveness of NSP and SVM programs, in terms of cost per HIV or HCV-case averted or cost per quality adjusted life year (QALY).

## Supplementary Information

**Additional file 1. **Cost data and analysis spreadsheet.

## Data Availability

All data generated or analysed during this study are included in this published article including a supplementary Excel file.
